# Neural Networks Technique for Filling Gaps in Satellite Measurements: Application to Ocean Color Observations

**DOI:** 10.1155/2016/6156513

**Published:** 2015-12-27

**Authors:** Vladimir Krasnopolsky, Sudhir Nadiga, Avichal Mehra, Eric Bayler, David Behringer

**Affiliations:** NOAA Center for Weather and Climate Prediction, 5830 University Research Court, College Park, MD 20740, USA

## Abstract

A neural network (NN) technique to fill gaps in satellite data is introduced, linking satellite-derived fields of interest with other satellites and* in situ* physical observations. Satellite-derived “ocean color” (OC) data are used in this study because OC variability is primarily driven by biological processes related and correlated in complex, nonlinear relationships with the physical processes of the upper ocean. Specifically, ocean color chlorophyll-a fields from NOAA's operational Visible Imaging Infrared Radiometer Suite (VIIRS) are used, as well as NOAA and NASA ocean surface and upper-ocean observations employed—signatures of upper-ocean dynamics. An NN transfer function is trained, using global data for two years (2012 and 2013), and tested on independent data for 2014. To reduce the impact of noise in the data and to calculate a stable NN Jacobian for sensitivity studies, an ensemble of NNs with different weights is constructed and compared with a single NN. The impact of the NN training period on the NN's generalization ability is evaluated. The NN technique provides an accurate and computationally cheap method for filling in gaps in satellite ocean color observation fields and time series.

## 1. Introduction

The number of successful neural network (NN) applications in satellite remote sensing, meteorology, and oceanography has steadily increased over the last two decades [1, 2]. In these fields, NNs have been applied to address such problems as classification, feature extraction and tracking, pattern recognition, change detection, forward and inverse problems, and so forth. NNs also have been used to fill data gaps in measurement time series [3–5], while Peres et al. [6] used NNs to extend observation records.

NNs have also been applied in satellite remote sensing of ocean color (OC) (see references below). The “color” of the ocean is determined by the interactions of incident light with substances or particles present in the water because suspended particles will increase the scattering of light. In coastal areas, material in river runoff, resuspension of bottom material (sand, silt) by tides, waves, and storms, as well as biologically active components in the water column can change the color of near-shore waters. These components contain substances that absorb certain wavelengths of light, altering the optical signature. For example, microscopic marine algae (phytoplankton) have the capacity to absorb light in the blue and red regions of the spectrum due to the chlorophyll which enables photosynthesis. The underlying principle for remote sensing of ocean color (OC) is water with higher concentrations of phytoplankton (chlorophyll) is greener, while water with lower concentrations of phytoplankton is bluer [7]. Ocean color data is a vital resource for operational forecasting, oceanographic research, and earth sciences, along with a wide variety of related applications [8].

Satellite-derived OC fields are essential for numerical prediction applications, enabling numerical models to address a biophysical feedback process that is particularly important to coupled ocean-atmosphere modeling. As a new capability, integrating/assimilating near-real-time OC data into numerical ocean modeling improves forecast accuracy; consequently, a robust method needs to be developed for filling data gaps and providing the projected OC values needed to run the model into the future for predictions. The assimilation of OC data also drives/constrains the modeling of physical-biogeochemical processes that are the foundation for ecological forecasting; however, such models require continuous (gap-free) satellite ocean color fields for development, initialization, and data assimilation [8]. Thus, this work contributes a critical foundation for physical and biogeochemical modeling by providing continuous ocean color data and projected values. For the past three years, NOAA has been the developing capability to use near-real-time Visible Infrared Imaging Radiometer Suite (VIIRS) and other OC fields for its operational ocean [9, 10] and coupled seasonal forecast systems [11].

Multiple NN applications have been developed to solve forward and inverse problems in satellite ocean color remote sensing [12, and references there]. NNs also have been applied to merging OC information from multiple satellite missions [13]. In this work, we developed a new NN approach, which allows gaps (spatial and temporal) in satellite derived OC fields to be filled using physically related, but independently derived, satellite and* in situ* observations which provide physical information about the state of the upper layer of the ocean.

## 2. Methodology

### 2.1. Formulation of the Problem

Chlorophyll-a (Chl-a) concentration, a biological proxy for the intensity of photosynthesis derivable from ocean color observations, is affected by processes in the upper layers of the ocean of various spatial and temporal scales. Physical parameters characterizing the state of the ocean surface and upper mixed layer—temperature, salinity, and density—define the active physical background for associated biological processes; thus, variability of the physical background is responsible for a significant portion of the variability of entrained biological parameters. Accordingly, we can consider the OC, *Y* (in this case, the single parameter Chl-a), as a function or mapping of a vector of the ocean surface and upper mixed-layer state variables, *X*. This mapping can be symbolically written as

(1) $$\begin{array}{c} Y=M\left(\;X\;\right);\;X\in R^{n},\;\;Y\in R^{m}, \end{array}$$

where *M* denotes the mapping, *n* is the dimensionality of the input space, and *m* is the dimensionality of the output space.

This function/mapping is expected to be a complex nonlinear function because the variability of the physical parameters is transferred into the OC variability through a complex hierarchy of physical, chemical, and biological processes. Also, both the OC and ocean state data have finite spatial and temporal resolutions (provided on a grid with limited spatial resolution and averaged to daily temporal resolution); consequently, the physical and biological variability on scales finer than these resolutions appear as stochastic contributions to the OC, *Y*. Thus, the mapping between the OC, *Y*, and physical ocean variables, *X*, is a complex, nonlinear stochastic mapping,

(2) $$\begin{array}{c} Y=M\left(\;X,\varepsilon \;\right). \end{array}$$

The stochastic variable *ε* represents an uncertainty introduced into the OC, *Y*, due to unaccounted high-frequency small scale (subgrid) variability of physical, chemical, and biological processes. Also, all or a part of variables, constituting vectors *Y* and *X*, are observations, which have different levels of noise. This noise also contributes into the stochastic variable *ε*. Assuming that stochastic part of the mapping is additive, representation (2) can be simplified,

(3) $$\begin{array}{c} Y=M\left(\;X\;\right)+\varepsilon . \end{array}$$

It is noteworthy that the uncertainty *ε* is an inherent informative part of the stochastic mapping, containing important statistical information about the mapping.* Actually, the stochastic mapping is a family of mappings distributed with a distribution function. The range and shape of the distribution function are determined by the uncertainty vector ε*.

### 2.2. NN Emulation for the OC Mapping

Neural networks are very generic, accurate, and convenient mathematical models that emulate complicated nonlinear input/output relationships through statistical learning algorithms [14]. NNs can be applied to any problem that can be formulated as a mapping (input vector versus output vector dependence). The multilayer perceptron (MLP) with one hidden layer is a generic tool for approximating such mappings [2]. The simplest MLP NN analytical approximations use a family of functions like

(4) yq=aq0+∑j=1kaqj·tanh⁡bj0+∑i=1nbji·xi;q=1,2,…,m,

where *x*_*i*_ and *y*_*q*_ are components of the input and output vectors *X* and *Y*, respectively, and *a* and *b* are NN weights. Here, the hyperbolic tangent is used as an activation function. Equation (4) is also a mapping, which can approximate any continuous or almost continuous (with final discontinuities) mapping [2, 15]. Symbolically, it can be represented as *Y* = NN(*X*).

To train the NN that is emulating the mapping (1), an error function, *E*, is created,

(5) $$\begin{array}{c} E=\frac{1}{N}\overset{N}{\underset{i=1}{\sum }}{\left[\;Y_{i}-NN\left(\;X_{i}\;\right)\;\right]}^{2}, \end{array}$$

and minimized to find an optimal set of coefficients *a*_*ij*_ and *b*_*ij*_. However, for stochastic mapping (3), the training criterion should be modified as

(6) $$\begin{array}{c} E=\frac{1}{N}\overset{N}{\underset{i=1}{\sum }}{\left[\;Y_{i}-NN\left(\;X_{i}\;\right)\;\right]}^{2}\leq \varepsilon^{2}. \end{array}$$

A single NN does not provide an adequate emulation/approximation of the stochastic mapping (3); therefore, an ensemble of NNs should be trained using criterion (6) [2]. If each NN member of the ensemble satisfies condition (6), this ensemble provides an adequate approximation for the stochastic mapping (3). Thus, to effectively account for subgrid scale effects and to reduce the impact of noise in NN simulated data (e.g., the Chl-a concentration), an ensemble of NNs was trained using criterion (6) and the average of this ensemble was used as the simulated OC, *Y*. In producing the ensemble of NNs to start the NN training, a slightly different initialization of NN weights, *a*_*ij*_ and *b*_*ij*_, was chosen for each NN ensemble member; thus, different NN ensemble members correspond to different local minima of the error function (5), all satisfying condition (6). This simplest approach was selected because the data have a significant level of uncertainty/noise. The magnitude of the uncertainty estimated in Section 4.1.2 (Table 2) shows that the basic ensemble approach allows us to obtain an approximation error of magnitude close to the magnitude of uncertainty in the data. In our opinion, this result shows that, consistent with the parsimony principle, the use of more sophisticated approaches is not justified in this case.

The ensemble was also used to improve the stability of the NN Jacobian [16], which is used below for a sensitivity study. The Jacobians of each *k*th NN ensemble member (*m* × *n* matrix of the first derivatives of the NN outputs over the input),

(7) $$\begin{array}{c} J_{k}={\left[\;\frac{\partial y_{q}}{\partial x_{i}}\;\right]}_{i=1,\ldots ,n}^{q=1,\ldots ,m}, \end{array}$$

were calculated and then averaged to calculate the mean Jacobian used for the sensitivity study below. Formally speaking, the Jacobian of the MLP NN (4) can be easily calculated using direct differentiation,

(8) ∂yp∂xs=∑j=1kbpj·1−tj2·ajs,

(9) tj=tanh⁡bj0+∑i=1nbji·xi.

However, calculating the derivative of any statistical model (including NN) is an ill-posed problem [2] which should be regularized. As shown by Krasnopolsky [16], the problem can be solved using an NN ensemble and calculating the Jacobian as an average of Jacobians of the NN ensemble members. This approach is used in this effort.

### 2.3. Selecting NN Inputs and Outputs

Selecting the emulating NN architecture includes selecting *n* NN inputs, *m* NN outputs, and the number of hidden neurons, *k*. For this study, we selected one output—chlorophyll-a concentration. The vector of inputs, *X*, was composed of two parts $X=\{\vec{a},\vec{b}\}$, where $\vec{b}$ is a vector of physical parameters, which includes satellite sea-surface elevation (SSH), sea-surface salinity (SSS), and sea-surface temperature (SST) and* in situ* Argo salinity (sal) and temperature (temp) vertical profiles. It can be expressed as

(10) $$\begin{array}{c} \vec{b}=\left\{\;\text{SSH},\text{SSS},\text{SST},\text{sal},\text{temp}\;\right\}, \end{array}$$

and vector $\vec{a}$ is a vector of auxiliary or meta variables or tracers configured as

(11) a→=yr,sin⁡τ,cos⁡τ,sin⁡lon,cos⁡lon,sin⁡lat,

where yr is the year, *τ* = 2*π*(*t*/365) (*t* equals the day of the year), and lon and lat are, respectively, longitude and latitude in radians.

Metadata are included in the input vector, *X*, to permit training a single NN (or single ensemble of NNs) that, given the input *X* for a particular location on the globe (lat and lon) at a particular moment in time (yr and *τ*), provides output (Chl-a concentration) for the same location and time. This NN is trained using records {*X*, *Y*} collected over several years at locations representing the entire globe. The trained NN (or NN ensemble), using the same weights, then is used for the entire globe for a long period subsequent to the training interval. Hence, each trained NN (a single NN or NN ensemble member) takes information from one grid point and produces a simulated value of OC (Chl-a) for that grid point at the corresponding time. The same single NN or NN ensemble then moves to the next grid point of the global grid, producing, in this way, a global field of OC (Chl-a). The results presented below are obtained with the input vector *X*, comprising $\vec{a}$ (11), including all metadata variables, and $\vec{b}$ (10), including three surface variables plus variables representing seven upper layers of sal and temp from Argo profiles. Thus, in this study, the NNs emulating the OC mapping each have 23 inputs and 1 output.  Table 1 lists these variables and their units, as well as the output parameter.

In the following assessment, an optimal set of inputs will be determined, specifically which parameters to include in (11) and the number of upper layers established from Argo profiles to include in (10). The accuracy of approximation and the correlation coefficient between NN-generated values and observed OC (Chl-a concentration) will be used as major indicators of the significance of the various inputs.

## 3. Data

### 3.1. Raw Data

The OC (chlorophyll-a concentration) data used in this study are from the Joint Polar Satellite System (JPSS) Visible Infrared Imaging Radiometer Suite (VIIRS). The VIIRS chlorophyll-a global fields are composited daily and interpolated from the 9 km resolution provided by NASA [17] to a global 1° × 1° latitude/longitude resolution.

Temperature and salinity profiles for the top 75 m of ocean water from Argo float data, a collaborative international partnership program for measuring upper ocean temperature, salinity, and currents in the Earth's oceans, were obtained from the International Pacific Research Center at Hawaii as gridded global Argo monthly mean data interpolated to daily values (1° × 1° latitude/longitude resolution) [18]. Daily global satellite SSH values [19] and daily global satellite SST, interpolated to 1° × 1° latitude/longitude grids, were acquired from NOAA [20]. NASA's Physical Oceanography Distributed Active Archive Center (PODAAC) provided SSS (bias adjusted, version 3, gridded) data from the Aquarius mission [21, 22]. Since the SSS fields do not have global coverage daily, the fields were composited to obtain daily coverage on a 1 by 1-degree global grid.

The aforementioned satellite and* in situ* observations are well documented and available in near-real time; however, the Aquarius mission recently ended (June 2015) due to equipment failure. These satellite and* in situ* data are interpolated to the same global one-degree latitude-longitude grid and are available at daily temporal resolution for the period 2012 through 2014.

### 3.2. Data for NN Training and Validation

The first two years (2012 and 2013 or 730 days) of daily data (~20,000,000 grid points or records) were selected for NN training and test. The data were split into training and test sets (~10,000,000 grid points or records each). Every second data record or grid point was selected for training, with the remaining records reserved for testing. Each record within the training and test sets consists of two vectors, input vector *X* (10, 11) and output vector *Y* (which actually is a scalar value in this study) at a particular grid location at a particular time (day). The data for 2014 (365 days) were reserved for set for validating trained NNs and estimating predictive (generalization) skill. For better understanding of the generalization skill of the OC NN approximation, the data was additionally partitioned with the 2012 data alone selected for training and test sets (~5,000,000 records each), using the 2013 and 2014 data for validation.

## 4. Results

### 4.1. The Accuracy of Approximation

This study determines the “optimal” architecture for the emulating NN using an early stopping method, evaluating the level of uncertainty in the data, and comparing performances of single NN and the NN ensemble. For this assessment, 2012 and 2013 data are used for training and test set, with 2014 data used for validation.

#### 4.1.1. Selecting the Number of Hidden Neurons

**Figure 1:**
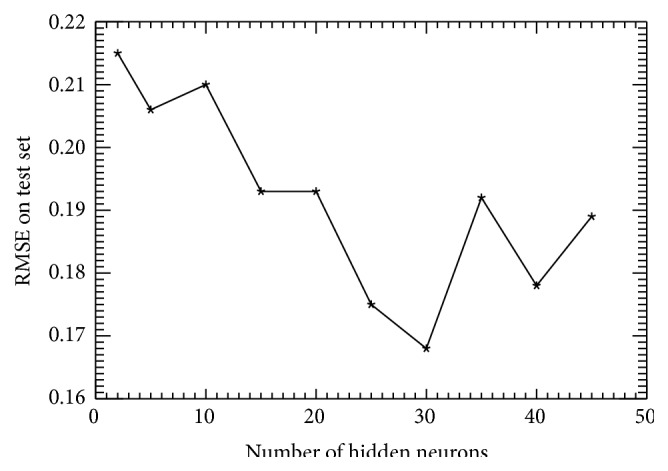
Chlorophyll-a neural network (NN) root-mean-square error (RMSE, mg/m^3^) as a function of the number of hidden neurons, *k* in (4), for an independent test set.

To evaluate the “optimal” size of the hidden layer (the number, *k*, of hidden neurons in (4)) supporting the previous selection of NN inputs and outputs, a set of ten NNs with 23 inputs and one output were trained, varying *k* from 3 to 45.  Figure 1 depicts the corresponding RMSE calculated for the independent test set.

From Figure 1, *k* = 30 is the “optimal” number of hidden neurons, because it provides the “best” approximation with the given training and test sets. At *k* > 30, the NN starts fitting the noise in the data; thus, in this case, the RMSE also provides an estimate for the uncertainty, *ε*, of order 0.2 mg/m^3^. Based on these considerations, *k* = 30 was selected for all NNs used to generate OC values in this study. Thus, almost all (except several special cases described in Section 4.1.2) single NNs and NN ensemble members trained and used in this study have the same architecture: 23 inputs, 30 hidden neurons, and 1 output.

#### 4.1.2. Estimating the Value of the Uncertainty

The above estimate of uncertainty, *ε* < 0.2 mg/m^3^, may be improved. The NN RMSE has several components and can be written as

(12) $$\begin{array}{c} RMSE=\varepsilon_{app}+\varepsilon , \end{array}$$

where *ε*_app_ is the approximation accuracy of the NN* per se* and *ε* is the uncertainty due to unaccounted fine scale processes, subgrid variability, and observation errors (Section 3.1). To better estimate the approximation accuracy of the NN simulating OC, we trained an NN with the same number of hidden neurons, *k* = 30, and one output, Chl-a concentration; however, this NN had only one input, the same Chl-a concentration. In other words, we trained an NN that emulated the identity mapping. Table 2 row 1 shows the approximation statistics for this NN, noting an approximation error of order 0.002 mg/m^3^, which is a very small portion of the RMSE (12) when adding Chl-a, as an additional input to the previously selected 23 inputs (Table 2, row 4), and comparing Table 2 rows 2 and 4, the RMSE and correlation coefficient between NN output and observed data do not significantly change. Thus, practically the entire RMSE, approximately 0.18 mg/m^3^ (Table 2 row 2), can be attributed to uncertainty due to subgrid processes and observation noise in the 23 inputs. Employing an NN ensemble does not materially change the estimate (Table 2 row 3).

**Figure 2:**
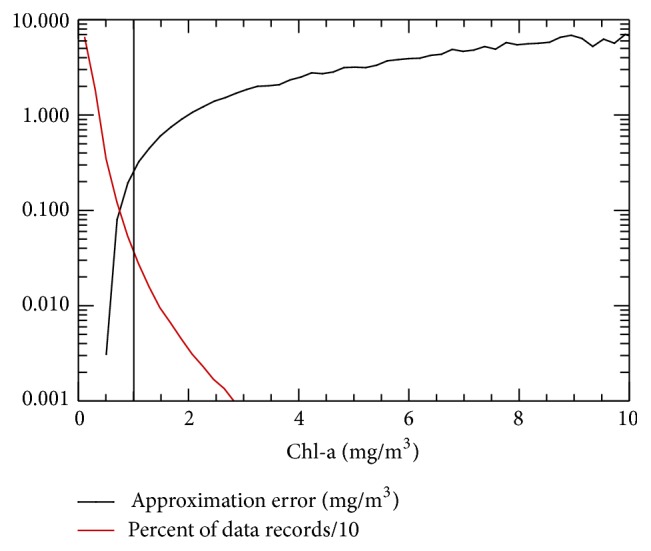
RMSE (black) and percentage (divided by 10) of data (red) as functions of Chl-a concentration. Vertical line shows Chl-a concentration = 1.0 mg/m^3^. There is only about 0.2% of data with concentrations greater than 1.0 mg/m^3^.

It is clear from physical considerations that the level of observation noise (errors) and subgrid uncertainty are higher at higher values of Chl-a variability (Figure 2). Higher levels of noise occur in coastal areas due to local subgrid processes. With higher Chl-a concentrations in coastal regimes, satellite observation errors are often higher and the accuracy of the retrieval algorithm is lower at higher levels of Chl-a, because there are few of* in situ* observations available for the algorithm development.

#### 4.1.3. Bias and RMSE

Of the data sets examined here, less than one percent of the grid points have a Chl-a concentration greater than 1.0 mg/m^3^ (Figure 2); thus, there is insufficient data for training NN, precluding NN Chl-a estimates from achieving adequate accuracy at higher concentrations of Chl-a. Accordingly, we train our NNs employing the full data set; however, results are presented for both the full OC interval and, when it is appropriate, the case where data points with Chl-a concentrations greater than 1.0 mg/m^3^ (about 0.2% of data) are removed. Table 2 rows 5 through 7 show the error statistics and correlation coefficients for NN cases presented in Table 2 rows 2 through 4, but with data records having Chl-a concentrations greater than 1.0 mg/m^3^ removed. For Chl-a concentration less than or equal to 1.0 mg/m^3^, the data uncertainty is significantly smaller (order 0.11 mg/m^3^ versus 0.18 mg/m^3^) and the correlation between the NN simulated OC and observed OC is significantly higher (0.72 versus 0.67).

The NN ensemble (Table 2 row 6) provides a significant RMSE and correlation coefficient improvement over those of the single NN (Table 2 row 5).

**Figure 3:**
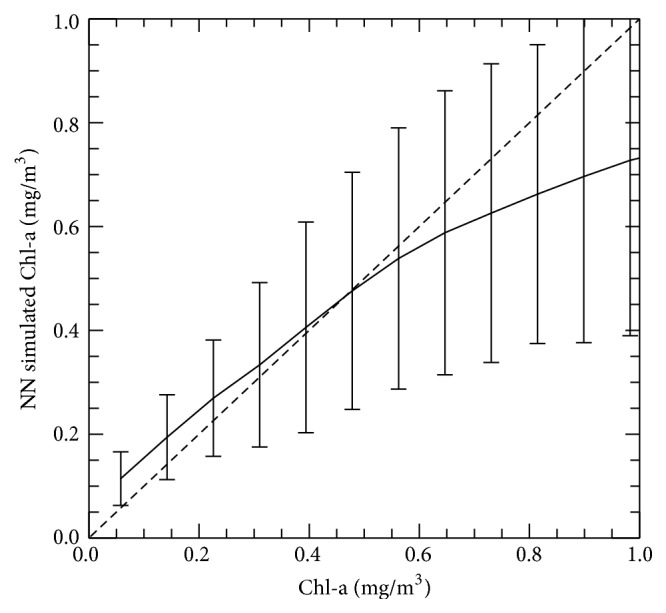
Binned scatter plot for NN-simulated versus observed ocean color (chlorophyll-a) data for chlorophyll concentrations less than or equal to 1.0 mg/m^3^; bars show the standard deviation of data in each bin.

**Figure 4:**
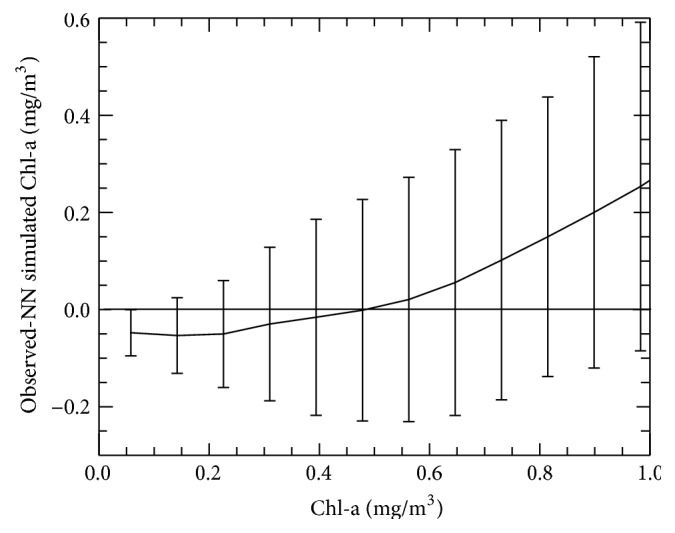
Binned dependence of approximation error (bias) on the value of chlorophyll-a concentration; bars show the standard deviation in each bin.

Figure 3 compares NN simulated versus observed Chl-a concentrations for Chl-a concentrations less than or equal to 1.0 mg/m^3^.  Figure 4 shows binned dependence of NN error (bias) on the value of Chl-a. In both figures, the standard deviation bars for each bin reflect the level of noise in the data. For Chl-a concentrations less than 0.5 mg/m^3^, the NN values have a small negative bias, while, for Chl-a concentrations above 0.5 mg/m^3^, the NN values have a small positive bias; however, the magnitudes of these biases are on order of the level of the data uncertainty.

#### 4.1.4. Performance of NN Ensemble

Finally, an ensemble consisting of six NNs ensemble members, with all six ensemble members having the same architecture (23 inputs : 30 hidden neurons : 1 output), was trained on the full NN training set using different initial values for NN weights, *a*_*ij*_ and *b*_*ij*_, (4). Thus, different NN ensemble members correspond to different local minima of the error function (5).  Table 3 displays the ensemble member and the ensemble average performances for Chl-a concentrations less than or equal to 1.0 mg/m^3^, showing that the ensemble mean has higher correlation between the NN output and the VIIRS observations and lower RMSE than any of the individual ensemble members. The ensemble mean clearly outperforms all of the individual ensemble members, suggesting that random noise may be contaminating the input and/or observation streams.

### 4.2. Evaluation of NN Prediction Capabilities

#### 4.2.1. Prediction Accuracy

**Figure 5:**
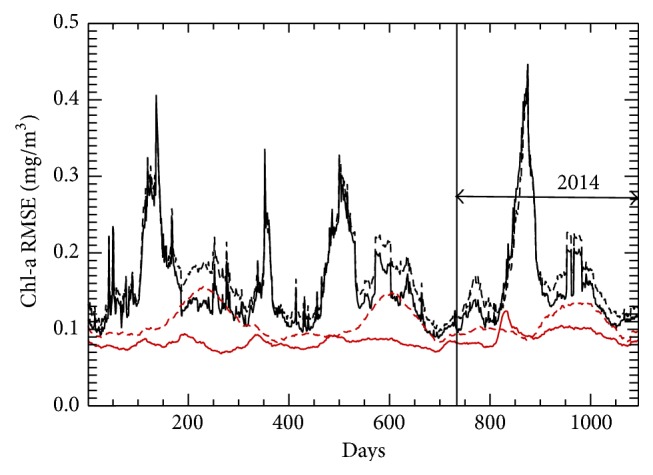
Three-year time series of global mean NN chlorophyll-a RMSE: black curves represent results from the full data set; red curves represent results when Chl-a values exceeding 1.0 mg/m^3^ have been removed (about 0.2% of data); solid lines show ensemble means and dashed lines depict values produced by an individual NN. The validation period (2014) is indicated.

Trained NNs and an NN ensemble have been applied to the data sets for 2012–2014 to produce Chl-a fields and to calculate statistics for validating the NN fields against observed VIIRS Chl-a observations. In Figure 5, the time series (black) for global mean Chl-a RMSE from an individual NN (dashed line) and from the NN ensemble (solid line) are presented. Also presented in Figure 5 are the corresponding time series when using only records with Chl-a concentrations less than or equal to 1.0 mg/m^3^ (red). Figure 5 supports our conclusion that the major contributor of noise in the VIIRS Chl-a data is the small amount of data with Chl-a concentrations greater than 1.0 mg/m^3^. These high concentration points, about 0.2% of the data set, create a problem for retrieval algorithm development and make training the NN for Chl-a concentrations greater than 1.0 mg/m^3^ more difficult.

**Figure 6:**
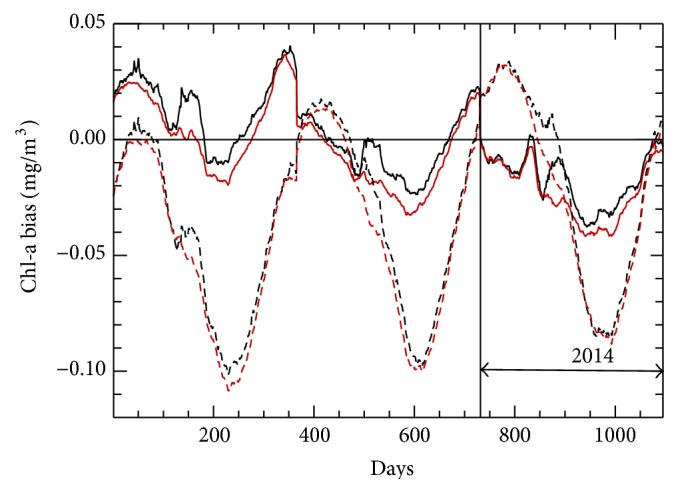
Three-year time series of global mean NN chlorophyll-a bias, referenced to VIIRS observations (VIIRS-NN values): black curves represent results from the full data set; red curves represent results when Chl-a values exceeding 1.0 mg/m^3^ have been removed (less than 1% of data removed); solid lines show the ensemble mean bias and dashed lines depict the mean bias produced by an individual NN. The validation period (2014) is indicated.

The mean biases (Figure 6) are very small, of order of the estimate for the level of noise in the data (Table 2 row 7). Figures 5 and 6 show a clearly pronounced annual cycle in the bias and RMSE when all data are used; however, removing the very small percentage of data, with Chl-a values exceeding 1.0 mg/m^3^, almost completely eliminates the annual cycle. Therefore, the small amount of data available with Chl-a concentrations greater than 1.0 mg/m^3^ is insufficient to train NN for higher values of Chl-a. For lesser concentrations of Chl-a, NN ensemble is accurate enough to remove the annual cycle from the bias.

**Figure 7:**
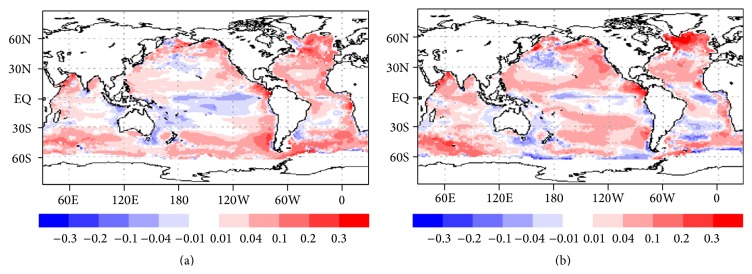
Spatial plot of global bias (Chl-a > 1 mg/m^3^ removed): (a) ensemble mean, (b) individual NN.

The spatial pattern of bias (Figure 7) has positive values in the equatorial Pacific Ocean and negative values in the equatorial Indian Ocean. As could be expected, large bias values are found at high latitudes and in shallow waters, for example, continental shelves and coastal regions.

**Figure 8:**
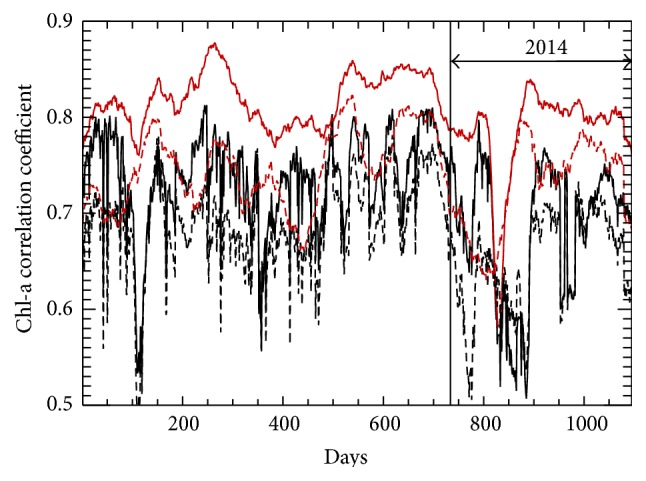
Three-year time series for NN Chl-a correlation referenced to VIIRS observations: black curves represent results from the full data set; red curves represent results when Chl-a values exceeding 1.0 mg/m^3^ have been removed (less than 1% of data removed); solid lines show the ensemble mean correlation and dashed lines depict the mean correlation produced by an individual NN. The validation period (2014) is indicated.

Figure 7 indicates that, when compared to results from an individual NN, the ensemble NN mean has lower bias in many regions of the global oceans, especially in the southwestern Indian Ocean, the North Pacific basin, and broadly in the Atlantic Ocean. The results for the tropical Pacific Ocean are mixed, with greater bias along the Equator and reduced biases throughout the higher latitudes of the Tropics.  Figure 8 shows the mean correlation (CC) is relatively stable and high throughout the validation period (~0.8), which is reassuring. The CC is lower and more variable for the cases where all data points are retained, suggesting that a few data points with Chl-a greater than 1.0 mg/m^3^ are responsible for notably degrading NN performance. Figures 5–8 indicate that an NN ensemble generally outperforms a single NN. These figures also indicate good generalization skill from NNs trained on two years of data.

#### 4.2.2. Evaluation of NN Generalization Skill

**Figure 9:**
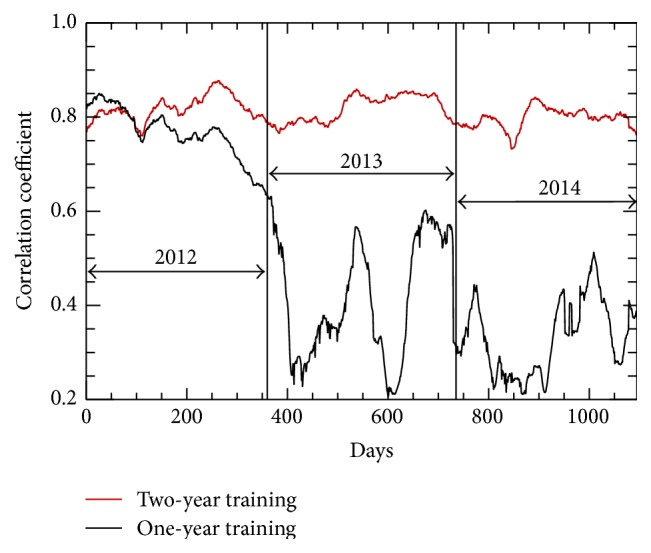
The correlation coefficient between the NN-generated chlorophyll-a and global VIIRS chlorophyll-a observed fields for 2012 through 2014: (red) a single NN trained with two years of data (2012-2013) and (black) a single NN trained with one year of data (2012).

To better evaluate generalization skill and the smallest training set required for accurate prediction of Chl-a fields, the second partition of data (Section 3.2), with only one year (2012) of training data, was used.  Figure 9 shows the correlation coefficient between the single NN-generated chlorophyll-a concentration fields and global VIIRS chlorophyll-a retrieval fields for 2012–2014 for two different single NNs: the first (red curve) corresponds to NN trained with two years of data (2012-2013) and second (black curve) to NN trained with one year of data (2012). As Figure 9 demonstrates the NN trained on one year of data very quickly loses its generalization skill beyond the training period (2012). The NN trained on two years of data preserves its generalization skill, generating data during the validation period (2014) of similar quality (in terms of correlation with observed data) to that produced during the training period (2012-2013). Similar depictions were obtained for bias and RMSE. Thus, we conclude that using a training data set of two years duration is sufficient to predict global OC fields for at least one year following the training period. These results demonstrate very good generalization skill by the NN, in terms of both spatial and temporal generalization.

#### 4.2.3. Preliminary Evaluation of NN Sensitivity

**Figure 10:**
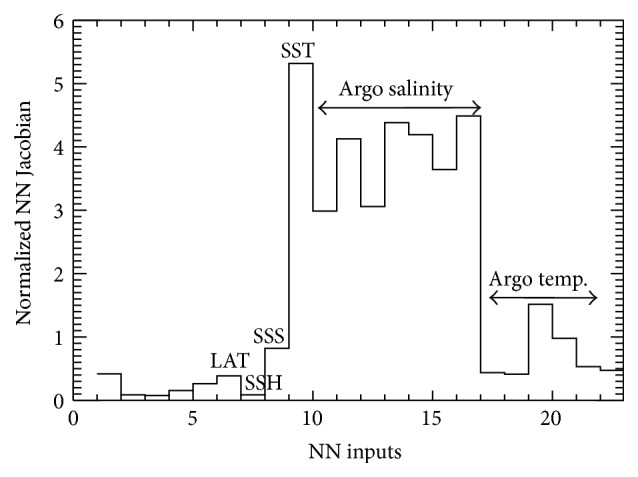
Linearized estimates (NN Jacobian Equation (9)) of the input relative contributions to the NN output, chlorophyll-a concentration.  Table 1 input: (1) year, (2) day (sine component), (3) day (cosine component), (4) longitude (sine component), (5) longitude (cosine component), (6) latitude, (7) sea-surface height, (8) sea-surface salinity, (9) sea-surface temperature, (10–16) Argo salinity profile, and (17–23) Argo temperature profile.

Using the NN Jacobian ((8) and (9)) for evaluating the relative contributions (importance) of the variables comprising the input vector, *X*, to the output vector, *Y*, the NN Jacobian for each NN ensemble member and then the mean Jacobian are calculated.  Figure 10 depicts the absolute value of the Jacobian vector components, giving an estimate of the significance of each NN input.  Figure 10 demonstrates that* the most important input is the SST, followed by the Argo surface and subsurface salinity fields.* The Argo surface temperature observations and satellite sea-surface salinity are less important than the aforementioned ones, perhaps because the satellite SST and Argo surface salinity observations already capture some of the variability of the other measurements.

## 5. Discussion and Conclusions

This work introduces a new NN application, using NNs to relate the biological parameter, Chl-a concentration, to physical processes of the upper ocean. This new NN maps satellite-derived surface parameters, for example, sea-surface temperature (SST), sea-surface height (SSH), and sea-surface salinity (SSS) fields, along with some* in situ* observations (upper layers of Argo salinity and temperature profiles), to Chl-a concentration. In other words, an NN empirical biological model for Chl-a is introduced in this paper. Ocean color (chlorophyll-a concentration) fields from NOAA's operational Visible Imaging Infrared Radiometer Suite (VIIRS) as well as NOAA SSH and SST fields and NASA Aquarius mission SSS fields were used. Observational data for 2012–2014 were spatially and temporally averaged and fitted to a 1° × 1° latitude/longitude grid for NN training, testing, and validation. Results were assessed using the mean error (bias), root-mean-square error (RMSE), and the correlations between observed and NN-generated chlorophyll-a concentrations. An ensemble of NNs with different weights was developed to reduce the impact of the noise in the data, as well as for calculating the relative significance of contributing inputs. Coarse spatial and temporal resolutions of the data limit the features resolved by the NN-generated OC fields. Global and mesoscale features are represented sufficiently well in the NN OC fields; however, resolving finer-scale features requires the NN to be trained with finer-resolution data.

This study demonstrates that employing an NN technique can provide an accurate, computationally cheap method for filling gaps in satellite observation fields and time series. It is noteworthy that an individual NN (or a single NN ensemble) is capable of generating OC fields for all global grid points, although an NN ensemble produces better results. This study demonstrated that training with at least two years of data is needed for sufficient skill to ensure that the accuracy of the NN prediction does not significantly degrade during the one-year validation period. These results demonstrate very good NN skill in terms of both spatial and temporal generalization. The NN approach successfully eliminates the systematic component of noise (bias). Employing an NN ensemble reduces the random component of the noise. When the small percentage of noisy data (less than one percent) is removed, the ensemble mean outperforms each of the ensemble members.

The mean Jacobian was used to evaluate the relative significance of the NN inputs, revealing that the daily SST is the most important input, closely followed by Argo monthly subsurface salinity profiles. The Argo monthly temperature subsurface signal moderately contributes to NN performance. We are planning to improve NN skills by (1) optimizing NN inputs, (2) retraining NN with accumulated new data, (3) introducing additional information (new input variables), and (4) employing higher resolutions for both the inputs and outputs. This NN system will be used to create consistent chlorophyll-a concentration time series across various ocean color satellite missions. The NN approach developed in this study and applied to filling ocean color satellite measurement gaps is a generic approach that can be applied to fill gaps in other satellite measurements. It is anticipated that this NN system will also be applied to other ocean color parameters important to numerical modeling, for example, the diffuse attenuation coefficient for photosynthetically active radiation (Kd_PAR_).

## Figures and Tables

**Table 1:** Inputs and outputs of emulating NNs.

Input #	Variable	Units	Input	Size
1	Year		yr	1
2	Day of the year		sin⁡((2 · day · *π*)/366)	1
3			cos⁡((2 · day · *π*)/366)	1
4	Longitude		sin⁡(lon)	1
5			cos⁡(lon)	1
6	Latitude		sin⁡(lat)	1
7	Sea surface height	m	SSH	1
8	Sea surface salinity	g/kg	SSS	1
9	Sea surface temperature	°C	SST	1
10–16	Argo salinity profile	g/kg	sal	7
17–23	Argo temperature profile	°C	temp	7

Output #	Variable	Units	Output	Size

1	Chlorophyll-a concentration	mg/m^3^	Chl-a	1

**Table 2:** Accuracy of approximation on independent test set.

	Type of NN (inputs : neurons : outputs)	Bias	RMSE	CC
1	1 : 30 : 1, (*ε*_app_)	4.*e* − 5	2.*e* − 3	1.00
2	23 : 30 : 1	−4.*e* − 2	1.76*e* − 1	0.67
3	Ensemble 6 NNs (23 : 30 : 1)	−2.*e* − 2	1.72*e* − 1	0.67
4	24 : 30 : 1	−4.*e* − 2	1.73*e* − 1	0.68
5	23 : 30 : 1 (Chl-a ≤ 1)	−2.*e* − 2	1.11*e* − 1	0.72
6	Ensemble (23 : 30 : 1) (Chl-a ≤ 1)	−2.*e* − 2	9.1*e* − 2	0.79
7	24 : 30 : 1 (Chl-a ≤ 1)	−3.*e* − 2	1.02*e* − 1	0.77

**Table 3:** Ensemble performance for Chl-a concentration ≤ 1.0 mg/m^3^.

Ensemble member #	RMSE (mg/m^3^)	Correlation coefficient
1	0.11	0.722
2	0.093	0.766
3	0.097	0.757
4	0.097	0.757
5	0.094	0.758
6	0.094	0.758
Ensemble average	**0.091**	**0.792**
